# Associations between stand structure and the predictability of multiple forest carbon pools in the transition zone between the Qinghai–Tibet and Loess Plateaus

**DOI:** 10.3389/fpls.2026.1935881

**Published:** 2026-10-21

**Authors:** Wenzhen Zong, Zengcao Ren, Lei Ling, Zhengni Chen

**Affiliations:** 1 Gansu Academy of Forestry, Lanzhou, China; 2 Key Laboratory of System Governance of Mountains, Rivers, Forests, Farmlands, Lakes, Grasslands and Deserts, National Forestry and Grassland Administration, Lanzhou, China; 3 Gansu Xinglong Mountain Forest Ecosystem Positioning Research Station, Lanzhou, China

**Keywords:** forest carbon density, random forest, spatial prediction, stand structure, transition zone

## Abstract

**Introduction:**

Complex mountain forests in the transition zone between the Qinghai–Tibet and Loess Plateaus are characterized by strong topographic heterogeneity and marked stand structural variation. These conditions may lead to differences in the predictability of forest carbon pools. However, the role of stand structure in shaping the predictability of multiple forest carbon pools remains insufficiently understood.

**Methods:**

In this study, we used field survey data from 90 plots (77 forest plots and 13 shrubland plots) collected in 2024-2025 in the Taohe National Nature Reserve, Gansu Province, together with vegetation indices, topographic, climatic, soil, and stand structural variables, to develop random forest models for multiple forest carbon pools. For plot-scale modelling, 77 forest plots were used, and soil carbon was assessed at 0–60 cm depth. We assessed the composition, predictability, and major predictors of biomass, soil, litter, deadwood, and ecosystem carbon densities.

**Results:**

The mean ecosystem carbon density was 306.79 Mg C ha^-1^, with soil carbon and biomass carbon accounting for 71.5% and 24.9%, respectively, suggesting that these two pools comprised the dominant carbon pools. Plot-scale random forest models showed clear differences in predictive performance among carbon pools. Biomass and ecosystem carbon densities were predicted with relatively high accuracy, with cross-validated R2 values of 0.766 and 0.659, respectively. Litter carbon density showed moderate predictability, whereas soil and deadwood carbon densities were poorly predicted. Biomass carbon density was primarily associated with stand volume proxy, tree height, and basal area proxy, whereas ecosystem carbon density was mainly associated with tree height, stand volume proxy, and stand age. Spatial random forest models showed relatively poor predictive performance.

**Discussion:**

These differences in predictive performance indicate that stand structural variables were closely associated with the predictability of biomass and ecosystem carbon densities. Overall, carbon density predictability in complex mountain forests showed clear component dependence. Component-specific modelling of multiple forest carbon pools helps reveal differences in their key predictors, predictive performance, and uncertainties associated with spatial extrapolation, and may provide useful information for forest carbon monitoring, spatial pattern characterization, and carbon management zoning in the Taohe Reserve.

## 1 Introduction

Forest ecosystems constitute a major component of the terrestrial carbon cycle and play an important role in regulating atmospheric CO_2_ concentrations, mitigating climate change, and maintaining regional ecological security (Fang et al., 2001; Pan et al., 2011). With intensifying global climate change and China’s “dual-carbon” strategy, accurate assessment of forest carbon stocks has become an important issue in forest ecology, carbon cycle research, and forest resource management (Guo et al., 2024; IPCC, 2019). Forest ecosystem carbon pools generally comprise biomass carbon, soil carbon, litter carbon, and deadwood carbon. These pools differ in formation mechanisms, turnover rates, and spatial heterogeneity (IPCC, 2019). Assessing multiple carbon pools is therefore important for characterizing the structure, allocation, and spatial variation of forest ecosystem carbon stocks.

Stand structure reflects forest developmental stage, resource competition, and canopy spatial organization, and is closely associated with forest carbon accumulation (Pretzsch, 2009; Pan et al., 2011). Structural attributes, including tree height, basal area, stand density, stand age, and stand volume, are closely linked to stand productivity, biomass accumulation, and organic matter return (Balderas Torres and Lovett, 2013; Khan et al., 2020). Compared with climate, topography, and soil background conditions, stand structural indicators may more directly represent forest growth status and carbon allocation. They may therefore explain variation in carbon density across multiple carbon pools more effectively (Zolkos et al., 2013; Sun et al., 2024). However, the relationship between stand structure and carbon storage varies among carbon pools. Biomass carbon is usually closely linked to tree height, basal area, and stand volume, whereas soil carbon, litter carbon, and deadwood carbon are also regulated by organic matter decomposition, soil carbon stabilization, decay stage, and local disturbance processes (Prescott, 2010; Schmidt et al., 2011; Cotrufo et al., 2013; Kawanishi et al., 2024). Thus, comparing the predictability of different carbon pools in relation to stand structure can help identify dominant controls and model applicability in complex mountain forests.

Conventional forest carbon assessment relies on field inventories, component sampling, allometric equations, and laboratory determination of carbon concentration. These approaches can yield accurate plot-scale estimates but are constrained by survey cost, sample size, terrain accessibility, and spatial representativeness, which can limit their use for continuous regional monitoring and spatial mapping (Sun et al., 2024). Multisource remote sensing products and gridded topographic, climatic, and soil datasets provide important auxiliary predictors for forest carbon modeling. However, these variables mainly represent canopy spectral conditions and environmental gradients. They cannot fully substitute for direct stand structural attributes such as tree height, basal area, and stand volume (Avitabile et al., 2016; Cheng et al., 2024; Yang et al., 2025). In complex mountain forests, it is therefore necessary to distinguish the explanatory role of plot-level structural variables from the extrapolation capacity of spatially continuous predictors. Plot-scale predictive performance should not be directly equated with spatial mapping accuracy.

Machine learning approaches, including random forest models, can capture nonlinear relationships and potential interactions between multisource predictors and forest carbon density. They have been widely used to estimate forest biomass, canopy height, and carbon stocks (Cheng et al., 2024; Zhang et al., 2024; Yang et al., 2025). Random forest models require relatively few assumptions about variable distributions and collinearity, and they can quantify predictor importance. They are therefore suitable for comparing the predictability of different carbon pools and identifying their dominant controls (Cheng et al., 2024; Sun et al., 2024). However, previous studies have mainly focused on aboveground biomass, aboveground carbon stocks, or total ecosystem carbon. Simultaneous modeling of multiple ecosystem carbon pools and comparison of their predictive performance remain limited (Avitabile et al., 2016; Sun et al., 2024; Yang et al., 2025). This limitation is particularly relevant in high-elevation complex mountain forests, where carbon pools may respond differently to stand structure, topographic hydrothermal conditions, soil properties, and local disturbance processes. Assessing a single carbon pool or directly modeling total ecosystem carbon may therefore obscure component-specific predictive boundaries and uncertainty sources.

The Taohe National Nature Reserve in Gansu Province lies in the transition zone between the Qinghai–Tibet and Loess Plateaus. It is an important water conservation area in the upper reaches of the Yellow River. The reserve is characterized by high elevation, steep slopes, fragmented terrain, and diverse vegetation types. The spatial distribution of forest carbon pools may therefore be jointly shaped by stand structure, topographic gradients, hydrothermal conditions, and soil background properties. Previous work has shown the potential of machine learning methods for retrieving forest structural parameters in the Taohe National Nature Reserve (Yang et al., 2025). However, the assessments of multiple forest carbon pools in this region remain limited. Few studies have compared the predictability of biomass, soil, litter, deadwood, and ecosystem carbon densities in relation to stand structure.

In this study, we integrated field survey data collected from 2024 to 2025 in the Taohe National Nature Reserve with stand structural parameters, remote-sensing vegetation variables, and topographic, climatic, and soil background predictors, to develop carbon-density models for multiple forest carbon pools. The specific objectives were to: (1) characterize the plot-scale patterns, composition, and variability of biomass, soil, litter, deadwood, and ecosystem carbon densities; (2) develop random forest models for different carbon pools using multisource predictors and evaluate differences in plot-scale predictability; (3) identify the relative contributions and marginal responses of stand structural and environmental variables across carbon pools, thereby clarifying the role of stand structure in shaping carbon pool predictability; and (4) conduct spatial extrapolation at the reserve scale using spatially continuous predictors to characterize broad spatial patterns and associated uncertainty of major carbon pools. This study aimed to characterize component-specific differences in carbon pool predictability and examine the associations between stand structural and carbon density predictability in complex mountain forests of the Qinghai–Tibet–Loess Plateau transition zone, thereby providing a basis for forest carbon monitoring, plot network optimization, and carbon-oriented management zoning in the Taohe Reserve.

## 2 Materials and methods

### 2.1 Study area

This study was conducted in the Taohe National Nature Reserve, Gannan Tibetan Autonomous Prefecture, Gansu Province, northwestern China (**Figure 1**). The reserve extends from 102°46′02″ to 103°44′40″E and from 34°10′07″ to 35°09′25″N. It lies on the northeastern margin of the Qinghai–Tibet Plateau and within the transition zone between the Qinghai–Tibet and Loess Plateaus, and represents an important water-conservation and biodiversity-protection area in the upper reaches of the Yellow River (Cheng et al., 2022). The reserve has a mean elevation of approximately 3360 m and a mean slope of 27.14°, reflecting typical mountainous terrain. It covers a total area of 287,760 ha, with forest cover of 45.24% (Lu, 2024). The region is characterized by a plateau continental monsoon climate, with a mean annual temperature of 5.1–5.8 °C and mean annual precipitation of 581–712 mm (Li et al., 2022). The vegetation belongs to the forest–steppe zone of the Qinghai–Tibet Plateau vegetation region, and shows clear aspect-related patterns. Spruce, fir, poplar, birch, and larch forests mainly occur on shaded and semi-shaded slopes, whereas shrub–grass communities and cypress forests are more common on sunny slopes (Li et al., 2022).

**Figure 1:**
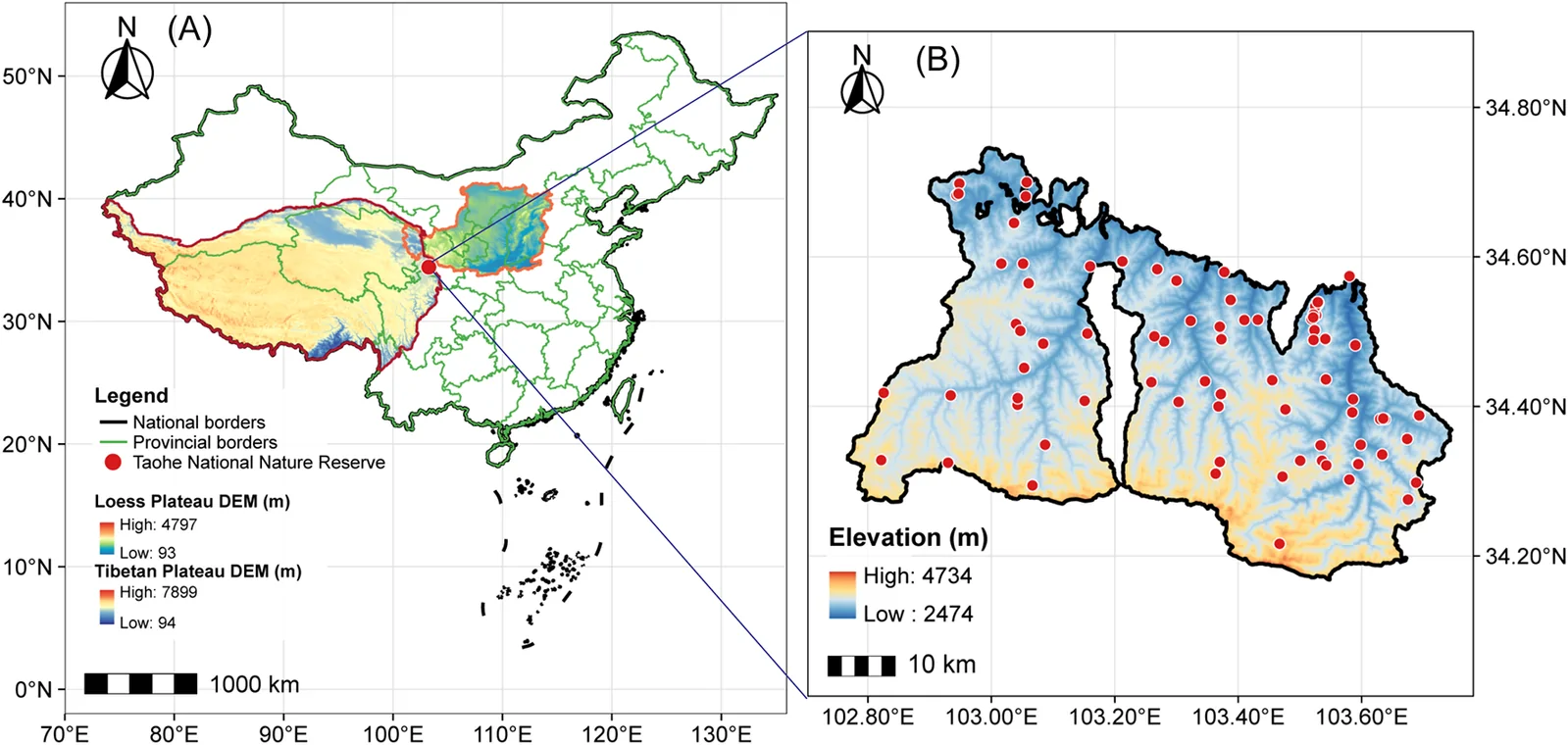
Location of the study area **(A)** and distribution of sample plots **(B)**.

### 2.2 Field survey and sample collection

Field surveys were conducted in the Taohe National Nature Reserve from June to August in 2024 and 2025. Sampling locations were selected to represent the major vegetation type, elevation gradient, and topographic conditions of the reserve, while site accessibility was considered as a practical constraint during field implementation. A total of 90 plots, comprising 77 tree-dominated forest plots and 13 shrubland plots. The same sampling design and plot size were used in both survey years, with the field surveys conducted over two years due to the large number of plots. Each plot covered 20 m × 20 m. For each plot, geographic coordinates, elevation, slope, aspect, stand age, dominant tree species, and shrub type were recorded where applicable. Biomass, litter, deadwood, and soil were surveyed to estimate biomass carbon, litter carbon, deadwood carbon, soil carbon, and ecosystem carbon densities.

For tree-dominated forest plots, all trees with a diameter at breast height (DBH) greater than 4 cm were measured, including DBH and tree height. Individual-tree biomass was estimated using species-specific allometric equations for northwestern China (Zhou et al., 2018). The biomass of individual trees within each plot was summed to obtain total tree biomass. Because understory shrubs and herbaceous plants accounted for less than 5% of total vegetation biomass, tree biomass was used as a proxy for total vegetation biomass at the plot level. Stand age was determined by counting annual rings in increment cores collected at breast height from representative dominant trees. For shrubland plots, the dominant shrub species, basal diameter, shrub height, crown width, and coverage were recorded. Crown width was measured along both east–west and north–south directions, and the mean value was used as the average crown width of each individual shrub or shrub clump. Shrub biomass was estimated using species-appropriate shrub allometric equations based on shrub species and field-measured variables (Xie et al., 2018). Plot-level shrub biomass was calculated as the sum of the biomass of all shrub individuals or clumps within each plot and converted to biomass per unit area.

For litter and deadwood sampling, three 1 m × 1 m litter quadrats were randomly established within each plot. All undecomposed and partially decomposed litter above the soil surface was collected. Representative subsamples were obtained by quartering and transported to the laboratory. In this study, woody debris included only coarse woody debris because fine woody debris was scarce and considered negligible. For all deadwood within each plot, the DBH and height of standing dead trees were measured. For downed logs, diameters at both ends and at the midpoint, together with length, were recorded (Zhu et al., 2017). Downed wood was classified into four decay classes based on its degree of decomposition (Zhu et al., 2017). Downed wood samples representing different decay classes were collected and transported to the laboratory. They were oven-dried to constant weight, weighed, ground, sieved, and analyzed for carbon concentration. The relationship between deadwood volume and dry mass was used to estimate plot-scale deadwood dry mass and carbon density (Zhu et al., 2017).

For soil sampling, sampling points were arranged within each plot using an S-shaped scheme. Soil samples were collected with a soil auger from three depth intervals: 0–20, 20–40, and 40–60 cm. For each depth interval, three replicate samples were collected from each plot and transported to the laboratory for soil organic carbon determination. In addition, one representative soil profile was excavated in each plot, and three undisturbed soil cores were collected from each depth interval using 100 cm³ cutting rings. The soil cores were oven-dried at 105 °C to constant weight to determine soil bulk density. The mean bulk density of the three cores was used for the corresponding soil layer.

### 2.3 Determination of carbon concentration

Litter and woody debris samples collected in the field were oven-dried at 65 °C to constant weight, and their dry mass was recorded. Dried plant samples for carbon concentration analysis were ground and passed through a 100-mesh sieve. Soil samples were air-dried, ground, and passed through a 2 mm sieve; gravel particles larger than 2 mm were collected and weighed. Subsamples were obtained by quartering, ground, and passed through a 100-mesh sieve for subsequent determination of organic carbon concentration. Carbon concentrations of plant and soil samples were measured using an elemental analyzer (Flash 2000 HT, Thermo Fisher Scientific, USA).

### 2.4 Determination and calculation of carbon densities

#### 2.4.1 Biomass carbon density

Based on the study region and tree species, species-specific allometric equations were used to calculate the biomass of different tree components, including leaves, branches, stems, and roots (Equation 1) (Zhou et al., 2018). Individual-tree biomass was calculated as the sum of component biomasses, and plot-level stand biomass was calculated as the sum of all individual-tree biomass within each plot.

(1) $$W=a{\left(DBH^{2}H\right)}^{b}$$

where *W* is the tree biomass; *DBH* is the diameter at breast height; *H* is the tree height; *a* and *b* are equation coefficients.

Biomass carbon density was estimated by multiplying biomass by a carbon conversion coefficient of 0.5 (Fang et al., 2021).

#### 2.4.2 Litter and deadwood carbon densities

Litter carbon density was calculated as the product of litter dry mass per unit area and the measured carbon concentration. Deadwood carbon density was calculated as the product of deadwood dry mass per unit area and the measured carbon concentration.

#### 2.4.3 Soil carbon density

Soil carbon density was calculated from soil organic carbon concentration, bulk density, and the thickness of each sampled soil layer. Soil organic carbon density was calculated separately for the 0–20, 20–40, and 40–60 cm layers and then summed to obtain plot-level soil organic carbon density (Equation 2).

(2) $$SCD=\sum_{i=1}^{n}(BD_{i}-ST_{i})\times h_{i}\times SC_{i}\times 0.1$$

where *SCD* is the soil carbon density (Mg C ha^-1^); BDi is the soil bulk density of layer i, including gravel (g cm^-3^); ST_i_ is the soil gravel content of layer i (g cm^-3^); hi is the soil depth of layer i (cm); and SCi is the soil C content of layer i (g kg^-1^).

#### 2.4.4 Ecosystem carbon density

Ecosystem carbon density was calculated as the sum of biomass carbon, litter carbon, deadwood carbon, and soil carbon density.

### 2.5 Stand structural variables

To characterize the effects of stand structure on different carbon pools, stand basal area proxy and stand volume proxy were further calculated. Stand basal area proxy was calculated from the arithmetic mean DBH and stand density (Equation 3).

(3) $$\text{Stand basal area proxy}=\text{Density}\times \text{π}\times {\left(\frac{\text{DBH}}{200}\right)}^{2}$$

where Basal area proxy is expressed as m^2^ ha^-1^; Density represents stand density (trees ha^-1^); DBH represents the arithmetic mean diameter at breast height (cm).

The stand volume proxy was calculated from stand basal area proxy and mean tree height (Equation 4):

(4) Stand volume proxy=Stand basal area proxy×Height

where stand volume proxy is expressed as m^3^ ha^-1^; Height represents mean tree height (m).

### 2.6 Extraction, selection, and model development of multisource predictors

To develop predictive models for forest carbon pools, we extracted five groups of predictors: stand structural variables, remote-sensing vegetation variables, topographic factors, climatic variables, and soil background properties. Stand structural variables were derived from plot surveys and included stand age, DBH, tree height, stand density, basal area proxy, and volume proxy. A total of 90 plots were surveyed, including 77 tree-dominated forest plots and 13 shrubland plots. Because tree-dominated forests and shrublands differ in community structure and in the representation of stand structural attributes, the plot-scale prediction models were developed using only the 77 tree-dominated forest plots with complete stand structural measurements, DBH, tree height, basal area proxy, and volume proxy were all derived from field-based plot measurements. In contrast, the spatial prediction models used all 90 plots to improve sample representativeness for reserve-scale spatial mapping, and only included predictors available for all plots.

Spatial environmental predictors were mainly obtained and preprocessed using the Google Earth Engine (GEE) platform. These predictors included remote-sensing vegetation indices, topographic factors, climatic variables, soil background properties, and forest structural proxy variables. All spatial datasets were clipped to the boundary of the Taohe National Nature Reserve and harmonized by projection, resampling, and raster alignment. For plot-level extraction, a 50 m buffer was generated around the coordinates of each plot to reduce the influence of geolocation uncertainty and local pixel-level variability, and the mean value of each predictor within the buffer was extracted.

Remote-sensing vegetation predictors included MODIS leaf area index (LAI), Sentinel-2 spectral bands, and Sentinel-2 vegetation indices. LAI was derived from the MCD15A3H product, from which growing-season mean values were extracted. Sentinel-2 data were obtained from the Sentinel-2 Surface Reflectance Harmonized product, using images acquired during the growing season from May to September 2024. Low-quality pixels, including no-data pixels, saturated pixels, cloud shadows, clouds, cirrus, and snow/ice, were removed using an S2 Cloud Probability threshold of <40 and the Scene Classification Layer (SCL). After cloud masking, growing-season median values were extracted for bands B2, B3, B4, B5, B6, B7, B8, B8A, B11, and B12. Among the Sentinel-2-derived vegetation indices calculated as candidate predictors, the normalized burn ratio (NBR) and normalized difference vegetation index 67 with band6 and band7 (NDVI67) were retained in the final models through the variable-selection procedure applied to the present dataset, following the calculation approach described in the literature (Yang et al., 2025). Sentinel-2 spectral and index variables were extracted at 20 m spatial resolution, whereas most other environmental variables were extracted at 250 m resolution.

Topographic predictors were derived from the SRTM DEM and included elevation, slope, aspect, Northness, and Eastness. Climatic predictors were obtained from the TerraClimate dataset and included mean annual temperature (MAT), mean annual precipitation (MAP), potential evapotranspiration (PET), and aridity index (AI = MAP/PET), using data from 2024. Soil background predictors were obtained from SoilGrids and included clay content, soil pH, and soil bulk density in the 0–30 cm layer. The 0–30 cm soil properties were calculated as thickness-weighted means of the 0–5, 5–15, and 15–30 cm layers and used as spatial predictors representing the surface soil environment at the plot level. It should be noted that the field-measured SOC at 0–60 cm was used as the response variable, and thus its sampling depth did not exactly correspond to that of the 0–30 cm soil background predictors. Elevation, slope, and 0–30 cm soil bulk density were further corrected using plot-measured values. Canopy cover was derived from the MODIS Vegetation Continuous Fields (VCF) product (MOD44B) and was used as a predictor in both the plot-scale and spatial models.

Before model fitting, all candidate predictors were examined for missing values and outliers. Multiple candidate predictor sets were defined *a priori* based on their ecological relevance, data reliability, and spatial continuity. These predictor sets were established prior to model evaluation and were not subsequently modified in response to model performance or predictions. Within each candidate set, predictors were ranked according to the absolute value of their correlation coefficient with the corresponding carbon pool and sequentially introduced into the random forest regression model in descending order of correlation strength. Importantly, correlation analysis was used only to determine the order of predictor entry and did not serve as a criterion for the inclusion or exclusion of individual predictors. The optimal number of predictors within each candidate set was determined based on predictive performance assessed by 5-fold cross-validation repeated 5 times. The best-performing model from each candidate predictor set was then compared, and the predictor set yielding the highest predictive performance was selected as the final predictor set. Pairwise Pearson correlation coefficients were calculated among the predictor variables included in the best-performing random forest models to examine their correlation structure and potential collinearity. Two types of models were developed: plot-scale prediction models and spatial prediction models. The plot-scale prediction models used both field-measured stand structural variables and spatial environmental variables. These models were used primarily to compare the predictability of different carbon pools and identify dominant predictors and predictive associations. The spatial prediction models used only spatially continuous raster-based variables and were used to generate reserve-scale maps of forest carbon density.

Random forest regression models were developed separately for biomass carbon, litter carbon, soil carbon, deadwood carbon, and ecosystem carbon. Each random forest model consisted of 1000 decision trees. The number of candidate variables considered at each node split (mtry) and the minimum terminal node size (min.node.size) were tuned. Model performance was evaluated using repeated 10-fold cross-validation with 10 repeats. Evaluation metrics included the coefficient of determination (R²), root mean square error (RMSE), mean absolute error (MAE), normalized root mean square error (nRMSE), mean prediction bias (Bias), and the ratio of performance to interquartile range (RPIQ). The optimal model was selected based on cross-validated predictive performance, with higher R² and RPIQ and lower RMSE, MAE, and nRMSE, and absolute Bias indicating better performance.

To evaluate the predictive advantage of random forest models over conventional linear models, multiple linear regression (MLR) models were developed as baseline comparisons. Each MLR model used the same predictor combination as its corresponding random forest model and was evaluated using the same repeated cross-validation procedure. The MLR results were used only as a reference for model comparison. Subsequent variable-importance analysis, partial dependence analysis, and spatial prediction were conducted using the optimal random forest models.

### 2.7 Model interpretation and spatial prediction

Based on the optimal random forest models, permutation importance was used to quantify the relative contribution of each predictor to model predictive performance. For comparability among predictors, the predictor with the highest permutation importance within each model was assigned a value of 100%, and the importance of the remaining predictors was expressed relative to this reference value. These standardized values indicate the relative predictive importance of individual predictors within the model and should not be interpreted as measures of independent explanatory power or causal effects. Partial dependence plots were generated for the main predictors to visualize their relationships with model-predicted carbon-pool values. Cross-validated predictions were used to plot observed versus predicted values and to assess how well the models captured plot-scale variation in carbon pools.

For spatial prediction, models based only on spatially continuous predictors were applied to raster datasets covering the reserve. Spatial distribution maps were generated for biomass, soil, litter, and ecosystem carbon densities. Deadwood carbon density was not included as a primary mapping target because of its high proportion of zero values and low spatial prediction stability. During spatial mapping, a forest mask derived from the 2021 land-use status database generated from the Third National Land Survey was applied to retain predicted values only within forested areas. Given the generally low cross-validation performance of the spatial prediction models, the results were used mainly to characterize relative spatial patterns of carbon pools at the reserve scale, rather than to provide high-precision pixel-level estimates of carbon density.

## 3 Results

### 3.1 Plot-measured characteristics of different carbon pools

Forest ecosystem carbon density in the study area ranged from 198.07 to 420.55 Mg C ha^-1^, with a mean value of 306.79 ± 53.58 Mg C ha^-1^ (**Figure 2A**). In terms of carbon-pool composition, soil carbon and biomass carbon were the dominant components of the ecosystem carbon pool. Soil carbon density was 219.36 ± 25.67 Mg C ha^-1^, accounting for 71.5% of ecosystem carbon, whereas biomass carbon density was 76.31 ± 40.32 Mg C ha^-1^, accounting for 24.9% (**Figure 2B**). By contrast, litter carbon and deadwood carbon contributed relatively little, with mean contributions of 3.4% and 0.2%, respectively (**Figure 2B**). The variation differed markedly among carbon pools. The coefficients of variation for biomass, soil, and litter carbon densities were 52.8%, 11.7%, and 44.1%, respectively. The coefficient of variation for deadwood carbon density reached 202.0% (**Figure 2C**), suggesting a highly heterogeneous spatial distribution. Ecosystem carbon showed a strong positive correlation with biomass carbon (Pearson’s r = 0.870, *P* < 0.01), which was stronger than its correlation with soil carbon (Pearson’s r = 0.580, *P* < 0.01).

**Figure 2:**
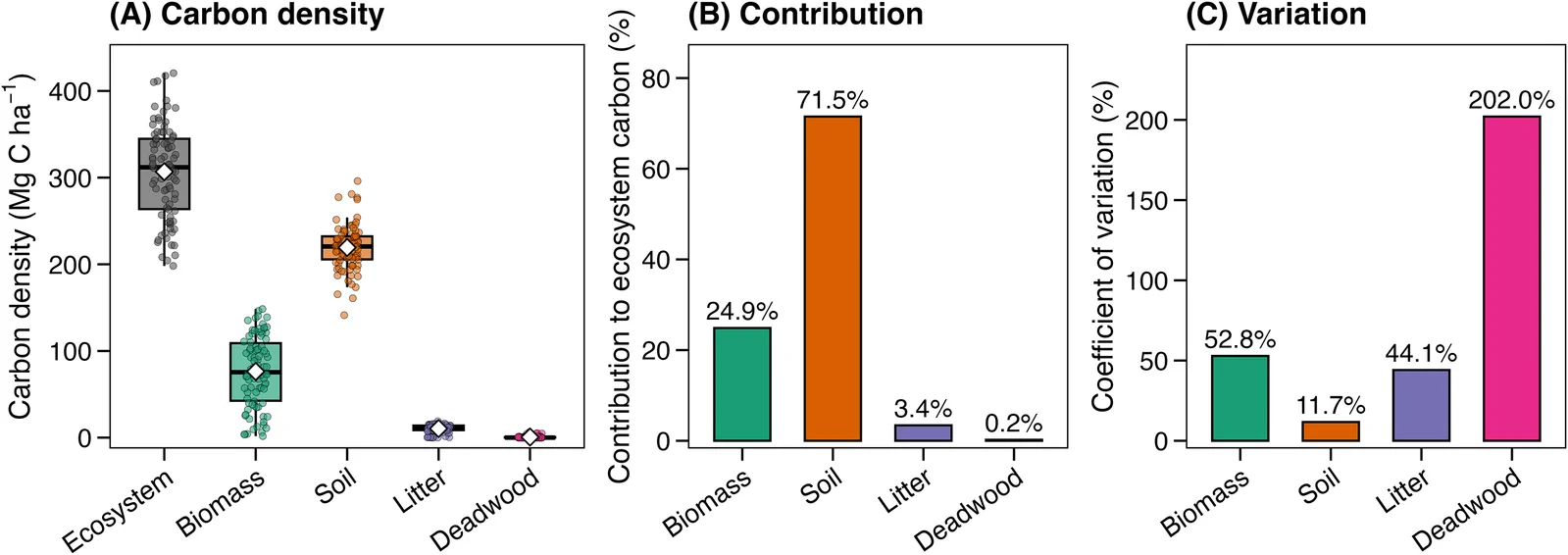
Carbon density **(A)**, relative contribution **(B)**, and variation **(C)** of the forest ecosystem and its component carbon pools in the Taohe National Nature Reserve. n=90 (77 forest plots and 13 shrubland plots).

### 3.2 Performance of plot-scale random forest models

The plot-scale predictive performance of the random forest models differed among carbon pools (**Table 1**). Overall, the biomass carbon and ecosystem carbon models performed relatively well, the litter carbon model showed moderate predictive ability, and the soil carbon and deadwood carbon models exhibited low cross-validated accuracy (**Table 1**; **Figure 3**).

**Table 1:** Cross-validated performance of plot-scale random forest models for carbon densities of the forest ecosystem and its component carbon pools in the Taohe National Nature Reserve.

Carbon pool	mtry	min node size	R^2^	RMSE	MAE	Bias	nRMSE%	RPIQ	Predictors
Biomass	3	3	0.766	16.696	13.499	0.465	19.254	3.275	Elevation; AI; Canopy cover; Age; Density; Height; Basal area; Volume
Litter	2	3	0.363	2.168	1.781	-0.006	18.091	1.929	LAI, Age, Density, Height, Basal area, Volume
Deadwood	1	3	0.103	1.351	0.881	0.068	172.206	0.549	Canopy cover, Volume, Northness, Height, Density, NDVI67, Age
Soil	5	10	0.103	23.327	17.551	0.081	10.532	1.049	Age, MAP, Soil pH, NBR, Canopy cover
Ecosystem	3	3	0.659	26.153	21.056	0.117	8.147	2.332	Elevation, AI, Canopy cover, Age, Density, Height, Basal area, Volume

Model performance was evaluated using repeated 10-fold cross-validation with 10 repeats. Mtry indicates the number of candidate predictors randomly selected at each split in the random forest model; min node size indicates the minimum number of observations in terminal nodes. R^2^, coefficient of determination; RMSE, root mean square error; MAE, mean absolute error; Bias, mean prediction bias; nRMSE, normalized root mean square error; RPIQ, ratio of performance to interquartile range, calculated as IQR/RMSE, where IQR is the interquartile range of the observed values. RMSE, MAE, and Bias are expressed in Mg C ha^-1^. Volume, stand volume proxy; Height, mean tree height; Basal area, stand basal area proxy; Density, stand density; AI, aridity index; LAI, leaf area index; Soil pH, soil pH in the 0–30 cm layer; Northness, the north–south component of aspect; MAP, mean annual precipitation; NBR, normalized burn ratio; NDVI67, the Sentinel-2 NDVI67 median.

**Figure 3:**
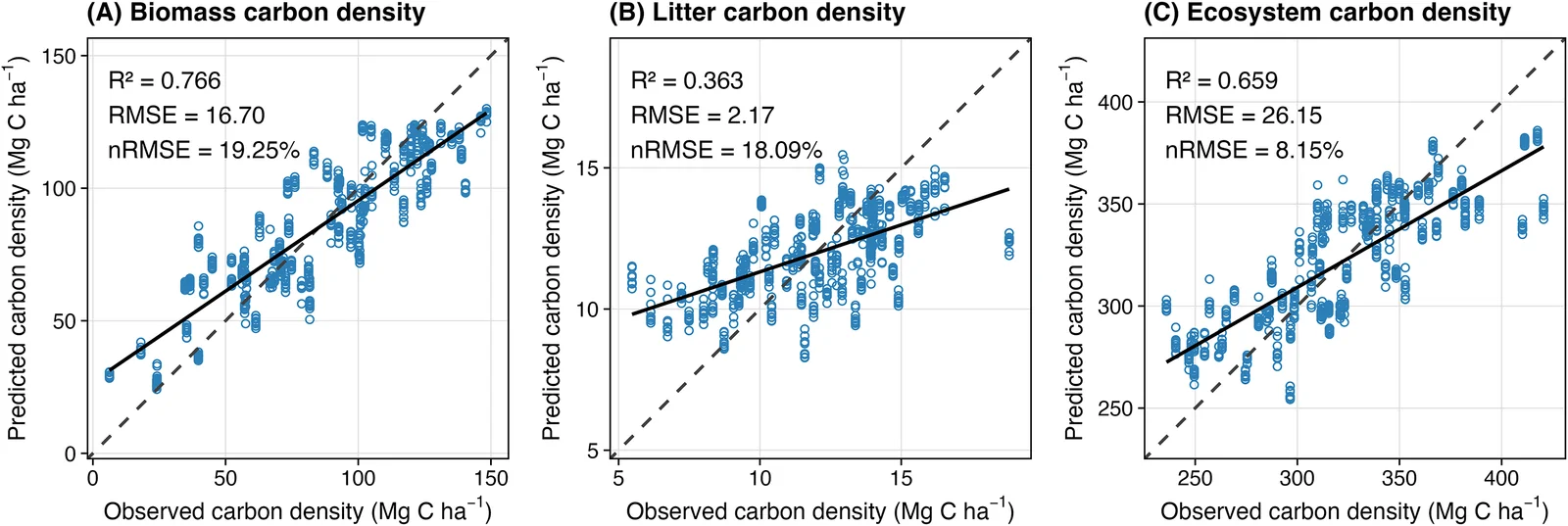
Cross-validated observed versus predicted carbon density from plot-scale random forest models for biomass carbon **(A)**, litter carbon **(B)**, and ecosystem carbon **(C)** in the Taohe National Nature Reserve. Dashed lines indicate the 1:1 reference line, and solid lines indicate fitted linear regression lines. R^2^, coefficient of determination; RMSE, root mean square error; nRMSE, normalized root mean square error.

The biomass carbon density model performed best among all carbon pools, with a cross-validated R² of 0.766, RMSE and MAE values of 16.696 and 13.499 Mg C ha^-1^, respectively, nRMSE of 19.254%, Bias of 0.465 Mg C ha^-1^, and RPIQ of 3.275. The small Bias suggested no evident systematic overestimation or underestimation. In the biomass carbon model, excluding tree height, basal area proxy, and volume proxy decreased the cross-validated R^2^ to 0.353 and increased the RMSE and nRMSE to 27.226 Mg C ha^-1^ and 31.398%, respectively, demonstrating the substantial contribution of stand structural variables to the predictive performance of the plot-scale biomass carbon model (**Supplementary Table S1**).

The ecosystem carbon density model also showed good predictive performance, with a cross-validated R² of 0.659, RMSE and MAE values of 26.153 and 21.056 Mg C ha^-1^, respectively, nRMSE of 8.147%, Bias of 0.117 Mg C ha^-1^, and RPIQ of 2.332. After excluding the stand structural variables, the ecosystem carbon model yielded a cross-validated R^2^ of 0.255, with RMSE and MAE of 38.051 and 28.978 Mg C ha^-1^, respectively, representing a substantial decline in predictive performance compared with the full model (**Supplementary Table S1**). The litter carbon density model showed moderate predictive accuracy, with a cross-validated R² of 0.363, RMSE and MAE values of 2.168 and 1.781 Mg C ha^-1^, respectively, nRMSE of 18.091%, and low Bias of −0.006 Mg C ha^-1^, indicating relatively stable prediction direction. By contrast, the deadwood carbon density model showed weak predictive ability, with a cross-validated R^2^ of 0.103, RMSE and MAE values of 1.351 and 0.881 Mg C ha^-1^, respectively, nRMSE of 172.206%, and RPIQ of 0.549. The soil carbon density model also had low cross-validated accuracy, with an R^2^ of 0.103, RMSE and MAE values of 23.327 and 17.551 Mg C ha^-1^, respectively, nRMSE of 10.532%, and RPIQ of 1.049 (**Table 1**; **Supplementary Figure S1**).

To evaluate the predictive advantage of random forest models relative to conventional linear models, multiple linear regression (MLR) models were constructed using the same predictor sets as the corresponding random forest models. MLR showed some predictive ability for biomass and ecosystem carbon densities, but its overall performance was lower than that of the random forest models, particularly for ecosystem carbon (**Supplementary Table S2**). Soil and deadwood carbon densities performed poorly in both MLR and random forest models, suggesting that the current predictor set did not adequately represent the variation in these two carbon pools. Therefore, subsequent variable importance analysis, partial dependence analysis, and spatial prediction were conducted using the random forest models.

### 3.3 Plot-scale drivers of carbon pools

Random forest variable importance analysis showed that the dominant predictors differed markedly among carbon pools, although biomass, litter, and ecosystem carbon densities were generally closely associated with stand structural variables (**Figure 4**). Biomass carbon density was mainly influenced by stand volume proxy, tree height, and basal area proxy, with relative importance values of 100.0%, 99.8%, and 45.1%, respectively, whereas density, age, AI, elevation, and canopy cover had relatively lower importance (**Figure 4A**). Partial dependence plots showed that biomass carbon density generally increased with increasing stand volume proxy, tree height, and basal area proxy, and tended to stabilize at higher values. The effect of basal area proxy on biomass carbon density showed a nonlinear pattern, with a continuous increase followed by stabilization (**Figure 5A**).

**Figure 4:**
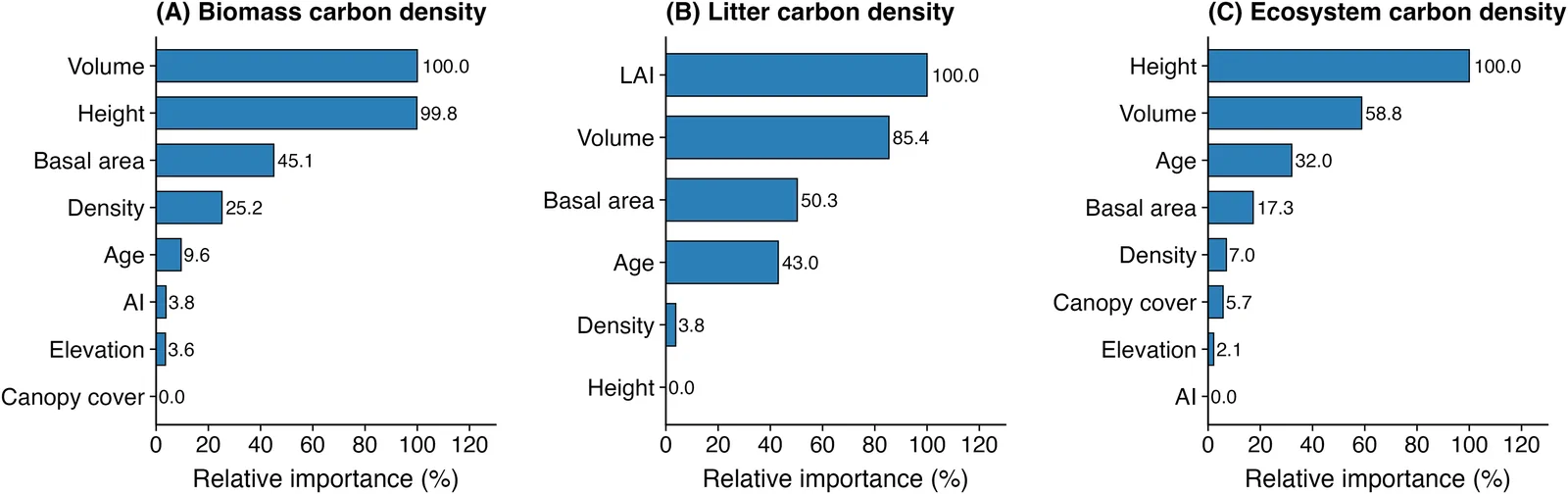
Variable importance derived from plot-scale random forest models for biomass carbon density **(A)**, litter carbon density **(B)**, and ecosystem carbon density **(C)** in the Taohe National Nature Reserve. Relative importance values were normalized within each model, with the most important predictor set to 100%. Volume, stand volume proxy; Height, mean tree height; Basal area, stand basal area proxy; Density, stand density; AI, aridity index; LAI, leaf area index.

**Figure 5:**
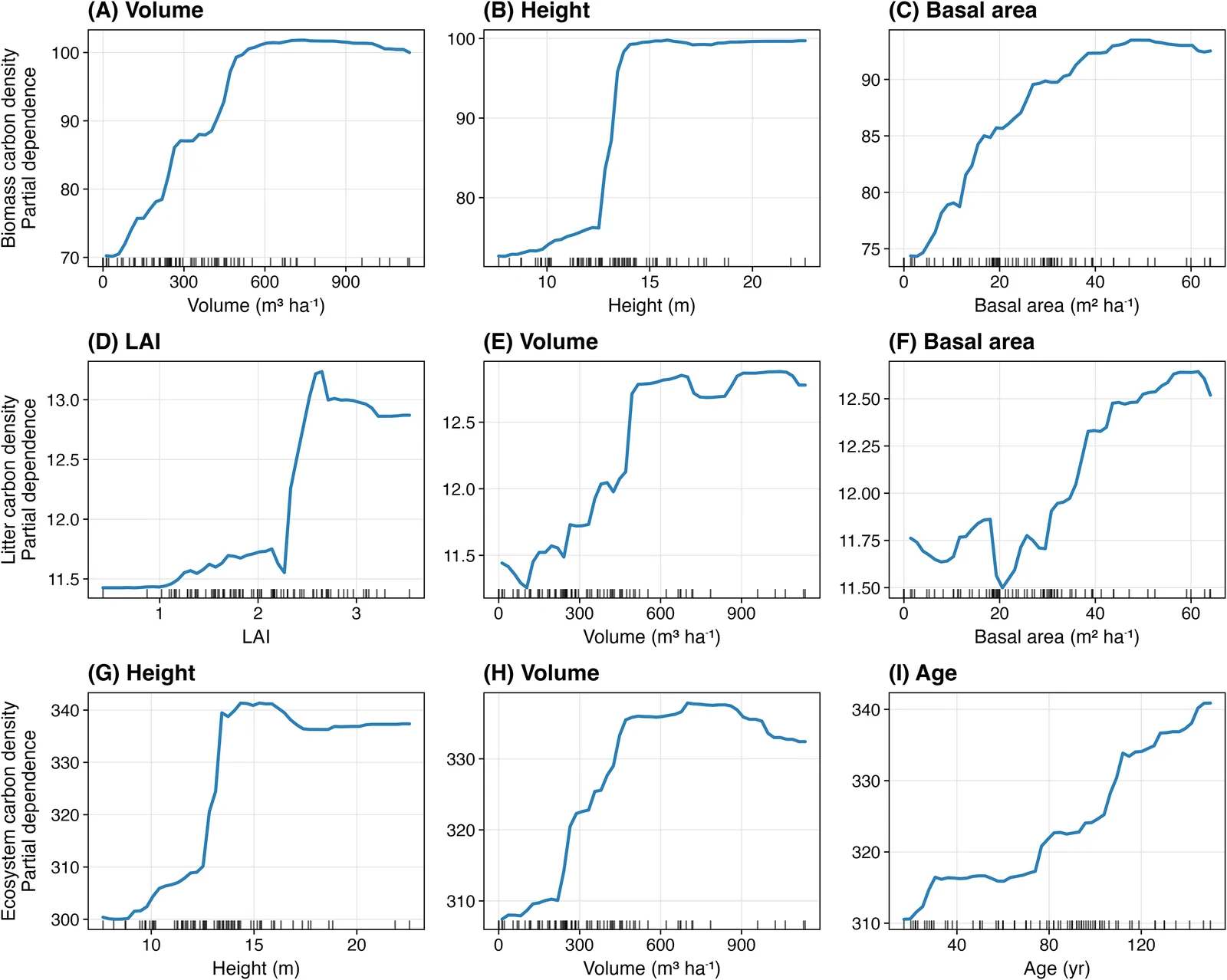
Partial dependence plots of the main predictors derived from plot-scale random forest models for biomass carbon density, litter carbon density, and ecosystem carbon density in the Taohe National Nature Reserve. **(A–C)** show the marginal effects of Volume, Height, and Basal area on biomass carbon density; **(D–F)** show the marginal effects of LAI, Volume, and Basal area on litter carbon density; and **(G–I)** show the marginal effects of Height, Volume, and Age on ecosystem carbon density. The rug plots along the X-axes indicate the distribution density of the observed predictor values used for model fitting. Volume, stand volume proxy; Height, mean tree height; Basal area, stand basal area proxy; LAI, leaf area index.

For the litter carbon density, LAI showed the highest relative predictive importance, with a relative importance of 100.0%, followed by stand volume proxy and basal area proxy, with relative importance values of 85.4% and 50.3%, respectively (**Figure 4B**). Partial dependence results showed that litter carbon density generally increased with LAI, followed by a slight decline and subsequent stabilization. Stand volume proxy and basal area proxy also showed positive nonlinear response patterns on litter carbon density (**Figure 5B**).

Ecosystem carbon density was strongly associated with tree height, stand volume proxy, and age, with relative importance values of 100.0%, 68.2%, and 34.2%, respectively, whereas basal area proxy, canopy cover, elevation, and density had relatively lower importance (**Figure 4C**). Partial dependence plots showed that ecosystem carbon density generally increased with increasing tree height, stand volume proxy, and age. These predictors showed nonlinear response patterns, with responses tending to stabilize at higher values. The effect of age on ecosystem carbon density showed a stepwise increasing pattern, with more pronounced increases at older stand ages (**Figure 5C**). Variable importance patterns for the deadwood carbon and soil carbon density models are presented in **Supplementary Figure S2**. Given the relatively low predictive performance of these models, these patterns were not further interpreted ecologically.

Overall, the plot-scale prediction of biomass, litter, and ecosystem carbon densities was mainly influenced by stand structural variables. The relationships between the main predictors and predicted carbon pool values were predominantly nonlinear.

### 3.4 Spatial distribution of carbon pools based on spatial prediction

The spatial prediction results provided model-derived spatial estimates of the overall distribution of different carbon pools in the Taohe National Nature Reserve exhibited varying degrees of spatial heterogeneity (**Figure 6**). The model-derived estimates suggested that high biomass, litter, and ecosystem carbon densities were mainly distributed in the central, northern, and parts of the eastern forested areas of the reserve. In contrast, relatively low estimated values were primarily observed in the southwestern region, along the southern margin, and in forested areas with low canopy cover. Soil carbon density showed a relatively narrow predicted spatial range, but relatively higher estimated values occurred mainly in the central-eastern region, whereas lower estimated values were distributed in the western and southern marginal areas. The estimated spatial distribution pattern of ecosystem carbon density was generally similar to those of biomass carbon and soil carbon.

**Figure 6:**
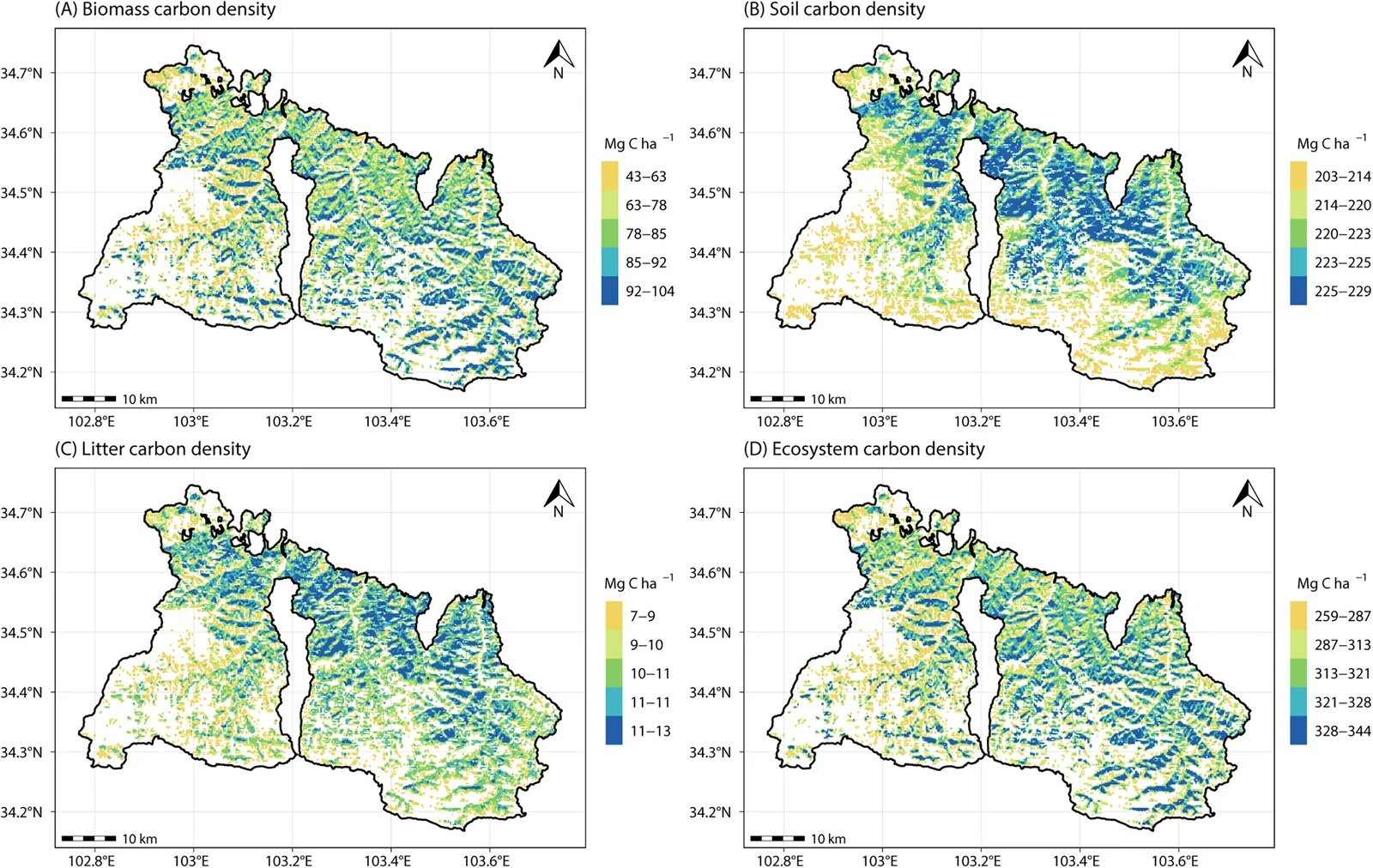
Spatial distribution of model-derived estimates of major forest carbon pools in the Taohe National Nature Reserve, showing biomass carbon density **(A)**, soil carbon density **(B)**, litter carbon density **(C)**, and ecosystem carbon density **(D)**. Carbon density is expressed in Mg C ha^-1^. The color gradients represent different carbon density classes, with yellow indicating lower values and blue indicating higher values.

Cross-validation results showed that the spatial prediction models had low overall predictive accuracy for all carbon pools (**Supplementary Table S3**; **Supplementary Figure S3**). Among them, the biomass carbon and ecosystem carbon models showed relatively higher predictive performance, but their cross-validated R² values remained low. The litter carbon and soil carbon models showed weak predictive ability. The cross-validated R² values of the four carbon pool models ranged from only 0.042 to 0.144, indicating that the spatially continuous predictors had limited explanatory power for plot-scale variation in carbon pools. Therefore, the spatial prediction results in this study were used mainly to characterize overall and relative spatial patterns at the reserve scale, rather than to provide high-precision pixel-level estimates of carbon density. Variable-importance results showed that relative importance of predictors varied among carbon pools in the spatial prediction models (**Supplementary Figure S4**). Given the low overall accuracy of the spatial prediction models, the variable-importance results were used only as auxiliary evidence for interpreting potential spatial associations.

## 4 Discussion

### 4.1 Composition and allocation pattern of ecosystem carbon density

The results of this study showed that forest ecosystem carbon density exhibited pronounced plot-scale spatial heterogeneity in the Taohe National Nature Reserve, and that the contributions of different carbon pools to ecosystem carbon density differed markedly. Carbon densities of soil and biomass accounted for 71.5% and 24.9% of ecosystem carbon density, respectively, representing the dominant components of the forest ecosystem carbon pool (**Figure 2B**). This allocation pattern is broadly consistent with the general understanding that forest ecosystem carbon pools are mainly composed of soil and living biomass. However, soil carbon accounted for a relatively large proportion of ecosystem carbon density within the 0–60 cm soil depth assessed in this study. Pan et al. (2011) reported that soil carbon to a depth of approximately 1 m and biomass carbon account for approximately 44% and 42% of global forest carbon density, respectively, and approximately 54% and 38% in temperate forests. Although the soil depth differed between studies, the relative contributions of soil and biomass carbon provide a useful reference for comparing broad carbon pool allocation patterns. The relatively large contribution of soil carbon in the present study may be associated with the high elevation, low temperature, and complex terrain of the Reserve. Low temperatures can suppress soil organic matter decomposition and microbial mineralization, thereby slowing soil carbon turnover (Davidson and Janssens, 2006; Bradford et al., 2016). Complex terrain can further modulate soil carbon inputs and stabilization by modifying soil hydrothermal conditions, vegetation composition, and erosion–deposition processes (Jobbágy and Jackson, 2000; Doetterl et al., 2015; Wiesmeier et al., 2019). Therefore, the forest ecosystems of the Taohe National Nature Reserve exhibited a pronounced soil-carbon-dominated pattern, suggesting that soil carbon is a key component influencing ecosystem carbon density in high-elevation temperate mountain forests.

### 4.2 Associations of stand structure with component-specific carbon density predictability

The plot-scale random forest results showed clear differences in predictive performance among carbon pools. The biomass carbon and ecosystem carbon density models showed relatively good predictive performance, the litter carbon density model showed moderate performance, whereas the soil and deadwood carbon density models showed relatively low performance (**Table 1**). These results suggest that the predictability of forest carbon density is strongly component-dependent, with the relative predictive importance of remote-sensing, environmental, and stand structural variables differing among carbon pools.

The relatively high predictive accuracy of the biomass carbon model was closely associated with stand structural variables. Variable importance and partial dependence results showed that stand volume proxy, tree height, and basal area proxy were among the most important predictors of biomass carbon density. However, correlated predictors can affect random forest variable-importance measures and complicate the interpretation of individual predictor contributions (Strobl et al., 2008). Biomass carbon density generally increased with increasing stand volume proxy, tree height, and basal area proxy, and tended to stabilize at higher values (**Figure 4A** and 5A). Because these variables are mathematically related to the estimation of biomass carbon, we further evaluated their influence on model performance using a sensitivity analysis. Sensitivity analysis showed that excluding tree height, basal area proxy, and stand volume proxy substantially reduced model performance (**Supplementary Table S1**), highlighting the importance of stand structural information for plot-scale biomass carbon prediction. At the same time, these structural variables are closely linked to the estimation of biomass carbon, and their inclusion may introduce a degree of mathematical dependence between the predictors and the response variable. Therefore, the relatively high predictive accuracy of the full model should be interpreted as the performance of an integrated plot-scale prediction model incorporating stand structural information, rather than as an independent measure of remote-sensing-based biomass prediction. This result is consistent with the ecological basis of forest biomass accumulation, as stand structural attributes provide important information for characterizing biomass development. It is also consistent with previous studies showing that canopy height, three-dimensional structural information, and stand volume-related information can improve the estimation accuracy of forest biomass and carbon density (Zolkos et al., 2013; Puliti et al., 2020; Sun et al., 2024).

The litter carbon density model showed moderate predictive ability, with LAI, stand volume proxy, and basal area proxy showing relatively high predictive importance (**Figure 4B**). LAI may reflect canopy leaf area and potential litter input, whereas stand volume proxy and basal area proxy reflect stand productivity and structural development. Litter carbon density generally increased with increasing LAI, but tended to stabilize or slightly decline at higher LAI levels (**Figure 5B**), indicating that litter carbon density is not determined by input quantity alone. Because litter carbon density is jointly regulated by litter input, decomposition rate, litter quality, and understory hydrothermal conditions (Prescott, 2010; Cotrufo et al., 2013), stand structural variables can explain only part of its variation and may not adequately represent litter decomposition and short-term dynamics.

Ecosystem carbon density was mainly influenced by tree height, stand volume proxy, and age (**Figure 4C**), suggesting that stand developmental status is closely linked to variation in ecosystem carbon density. Although soil carbon density was the largest component of ecosystem carbon density, accounting for 71.5% of ecosystem carbon, its predictive performance was relatively low (R^2^ = 0.103). In contrast, biomass carbon density accounted for only 24.9% of ecosystem carbon density but showed a stronger correlation with ecosystem carbon density (r = 0.870) than did soil carbon density (r = 0.580),. Sensitivity analysis further showed that excluding stand structural variables reduced the cross-validated R^2^ of the ecosystem carbon model to 0.255 (**Supplementary Table S1**), underscoring the important role of stand structural information in predicting ecosystem carbon density. These results indicate that the relatively high predictive performance of ecosystem carbon density was likely driven largely by the strong association between biomass carbon density and stand structure. Thus, the relative contribution of an individual carbon pool to ecosystem carbon density does not necessarily correspond to its predictability. In complex mountain forests, the total ecosystem carbon model should be regarded as an integrated assessment indicator, rather than a substitute for independent analyses of individual carbon pool.

By contrast, the soil and deadwood carbon density models had low accuracy (**Table 1**). Soil carbon formation and stabilization involve multiple processes, including plant inputs, root turnover, microbial transformation, mineral protection, and soil hydrothermal conditions (Jobbágy and Jackson, 2000; Schmidt et al., 2011; Lehmann and Kleber, 2015; Bradford et al., 2016). These processes are difficult to fully represent using the available remote-sensing indices, climatic variables, and limited soil background predictors, which may constrain the predictive performance of the soil carbon model. Previous studies have also indicated that forest SOC prediction often depends on local attributes such as soil moisture, soil type, texture, and vegetation type, whereas the explanatory power of remote-sensing and environmental covariates alone is limited (McBratney et al., 2003; Gomes et al., 2019; Hounkpatin et al., 2021).

Deadwood carbon density is associated with competition-induced mortality, windthrow, snow damage, pests and diseases, logging residues, and decay stage, which may contribute to a strongly patchy and stochastic spatial distribution (Harmon et al., 1986; Kawanishi et al., 2024). In this study, the coefficient of variation of deadwood carbon density reached 202.0% (**Figure 2C**), further indicating strong spatial heterogeneity. Conventional stand structural variables and remote-sensing vegetation indices therefore had limited capacity to explain its variation. Given the poor predictive performance of the deadwood carbon model (nRMSE = 172.206%) (**Table 1**), the relatively low predictive performance may reflect the complex and heterogeneous processes underlying deadwood dynamics, which are not fully captured by the available stand structural and remote-sensing variables. Accordingly, the relatively low predictive performance of the soil carbon and deadwood carbon models in this study likely reflects not only limitations of the modeling approach but also the complex processes underlying soil carbon stabilization and deadwood dynamics, which are insufficiently captured by conventional remote-sensing, environmental, and stand structural variables.

Overall, stand structure was closely associated with the predictability of biomass and ecosystem carbon densities, although its predictive importance varied among carbon pools. Biomass and ecosystem carbon densities showed relatively high predictability and stronger associations with stand structural variables, whereas litter carbon showed moderate predictability. In contrast, the low predictive performance of the soil and deadwood carbon models suggests that their variation was not fully captured by the available predictors. This limited predictability may reflect the complex processes underlying soil carbon stabilization and deadwood dynamics, contributing to greater uncertainty in their prediction.

### 4.3 Spatial extrapolation performation and methodological implications

Although biomass carbon and ecosystem carbon achieved relatively good cross-validated performance at the plot scale in this study, the spatial prediction models showed lower predictive accuracy (**Table 1**; **Supplementary Table S3**). This contrast highlights the challenges associated with extending plot-scale models to continuous spatial prediction. It also suggests that high plot-scale accuracy does not necessarily ensure stable spatial generalization, particularly in mountain forests with complex terrain and heterogeneous stand structure. The low accuracy of the spatial prediction models may be related to the lack of continuous raster support for key stand structural variables, including stand volume proxy, tree height, and basal area proxy, which contributed strongly to the plot-scale models (**Figure 4**). Spatial prediction instead relied primarily on remote-sensing indices, topographic factors, climatic variables, and soil background properties. Although these variables can represent canopy spectral conditions and environmental gradients, they cannot fully substitute for key three-dimensional structural information such as stand volume proxy, tree height, basal area proxy, and canopy vertical structure. Previous studies have shown that machine learning methods have potential for retrieving forest structural parameters in high-elevation complex mountain forests (Yang et al., 2025), and that integrating Light Detection and Ranging (LiDAR) and Synthetic Aperture Radar (SAR) structural information with optical remote sensing can improve forest biomass and carbon-stock estimation (Lefsky et al., 2002; Zolkos et al., 2013; Puliti et al., 2020; Sun et al., 2024; Wang et al., 2025). Therefore, the lower performance of the spatial prediction models may reflect, at least in part, the limited representation of forest three-dimensional structure by the available spatial predictors. Accordingly, their outputs should be interpreted as model-derived spatial estimates of the overall distribution of carbon pools rather than as high-precision pixel-level predictions.

Optical remote-sensing data have inherent limitations for carbon density prediction in complex mountain forests. First, optical vegetation indices are prone to signal saturation in high-biomass areas, which may obscure structural differences among high-volume stands. Second, topographic shadows, aspect effects, canopy complexity, and image quality can further affect spectral stability (Lu et al., 2016; Puliti et al., 2020; Xu et al., 2025). These factors may weaken the relationship between remote-sensing indices and actual carbon density, thereby increasing spatial extrapolation errors. Spatial prediction performance may also be affected by plot representativeness and terrain complexity. The Taohe National Nature Reserve has pronounced elevation gradients and fragmented terrain, and plot establishment was constrained by site accessibility. As a result, the plots may not fully cover the geographic and environmental feature space of the reserve. Sample clustering, incomplete feature-space coverage, and terrain-induced spectral variation can reduce the spatial generalization capacity of the models (Roberts et al., 2017; Araza et al., 2023; Meyer and Pebesma, 2021).

Furthermore, the spatially predicted soil carbon density exhibited a pronounced range contraction, with a substantially narrower range than that observed in the field measurements (**Figures 2**, **6**). Similar range-contraction patterns have been reported in random forest-based soil carbon density mapping, with low values tending to be overestimated and high values underestimated, causing predictions to shrink toward the center of the observed distribution (Liu et al., 2026; Hounkpatin et al., 2021). This range contraction may be attributed to the limited representation of extreme values in the training data, together with the limited ability of the available predictors to capture the spatial heterogeneity associated with these extremes, thereby constraining the model’s ability to reproduce the tails of the soil carbon density distribution. Consequently, the resulting soil carbon density map is more suitable for characterizing broad-scale spatial patterns than for accurately reproducing the full extent of local variability, and predictions of local extreme values should therefore be interpreted with caution.

Given these limitations, the spatial distribution maps generated in this study are more suitable for identifying relative spatial patterns of carbon pools at the reserve scale than for interpreting absolute carbon density values at the pixel scale. Future studies should further incorporate continuous three-dimensional structural data and adopt spatially structured validation and model-applicability assessment, together with feature-space coverage analysis and uncertainty mapping, to better evaluate the reliability of spatial carbon density predictions in complex mountain forests.

### 4.4 Research implications, limitations, and future perspectives

This study provides a framework for assessing multiple carbon pools in complex mountain forests and may inform carbon pool monitoring, plot network optimization, and carbon sink management zoning in the Taohe National Nature Reserve.

Several limitations remain. First, the Taohe Reserve has complex terrain and strong spatial heterogeneity in forest type, elevation, aspect, and hydrothermal conditions. The current number and spatial coverage of plots may not fully represent all environmental gradients within the reserve, which may limit model generalization. Second, key stand structural variables in the plot-scale models, such as stand volume proxy, tree height, and basal area proxy, lack continuous spatial raster support. Spatial extrapolation therefore relied mainly on remote-sensing indices, topographic, climatic, and soil background variables. These variables cannot fully represent three-dimensional stand structure, which may partly explain the lower accuracy of the spatial prediction models relative to the plot-scale models. Furthermore, the correlation-based ranking of predictors and their entry order were determined using the complete dataset prior to cross-validation, which may have introduced some optimism into the estimated model performance. However, because correlation analysis was used only to determine predictor entry order, whereas the final number of predictors and the optimal predictor set were determined based on cross-validated predictive performance, the resulting cross-validation estimates still provide a useful assessment of the predictive performance of the selected predictor sets. Additionally, hyperparameter tuning and model performance evaluation were conducted within the same cross-validation framework, and the random k-fold cross-validation did not fully account for the spatial clustering of sampling plots, which may have introduced some degree of optimistic bias in the estimated model performance. Nevertheless, future studies should employ nested cross-validation and spatial cross-validation, with predictor screening, variable selection, and hyperparameter tuning conducted within the training data of each outer fold to minimize potential information leakage and better evaluate model generalization under spatially structured sampling.

Future studies should strengthen long-term permanent plot monitoring, incorporate three-dimensional structural data from LiDAR, SAR, or unmanned aerial vehicle photogrammetry, and include process-related variables such as soil moisture, soil texture, microbial activity, mineral protection, decay class, and disturbance history. Spatial block cross-validation, feature-space coverage analysis, and uncertainty mapping should also be integrated to improve the reliability of spatial carbon density prediction in complex mountain forests. Overall, multisource predictors and random forest models can support multiple carbon pool monitoring in complex mountain forests, but their application should account for differences among carbon pools, limitations in structural data, and uncertainty in spatial extrapolation.

## 5 Conclusions

Based on field plot surveys, multisource remote-sensing and environmental predictors, and random forest models, this study evaluated the composition, predictability, and spatial extrapolation performance of multiple forest carbon pools in the Taohe National Nature Reserve. The results showed that the forest ecosystem carbon pool was dominated by soil carbon and biomass carbon, which accounted for 71.5% and 24.9% of ecosystem carbon density, respectively, indicating a pronounced soil-carbon-dominated pattern. At the plot-scale, the biomass and ecosystem carbon density models showed relatively good predictive performance, the litter carbon density model showed moderate performance, whereas the soil and deadwood carbon density models showed relatively low performance. These results indicate that model predictability differs clearly among carbon pools in complex mountain forests. Stand structural variables, including stand volume proxy, tree height, basal area proxy, and stand age, were among the most important predictors of biomass and ecosystem carbon densities, indicating that stand structural information provides valuable information for plot-scale carbon pool prediction. Spatial prediction models showed relatively low predictive performance, potentially reflecting limitations in the availability of continuous three-dimensional structural information and the effects of complex terrain. Accordingly, their outputs should be interpreted as model-derived spatial estimates of the overall distribution of carbon pools rather than as high-precision pixel-level predictions. Overall, separate modelling multiple carbon pools helps identify differences in their key predictors, predictive performance, and sources of uncertainty, and may inform forest carbon pool monitoring, carbon sink potential assessment, and management zoning in the Taohe Reserve.

## Data Availability

The original contributions presented in the study are included in the article/**Supplementary Material**. Further inquiries can be directed to the corresponding author.
