# Supplementary material for: Associations between stand structure and the predictability of multiple forest carbon pools in the transition zone between the Qinghai–Tibet and Loess Plateaus
Source: Front Plant Sci. 2026 Oct 21;17:1935881. doi: 10.3389/fpls.2026.1935881 (PMC13731113; doi:10.3389/fpls.2026.1935881)
Supplement: Supplementary file 1 [file DataSheet1.docx]

Supplementary Material

**Figure S1**. Cross-validated observed versus predicted carbon density from plot-scale random forest models for deadwood carbon (A) and soil carbon (B) in the Taohe National Nature Reserve. Dashed lines indicate the 1:1 reference line, and solid lines indicate fitted linear regression lines. R^2^, coefficient of determination; RMSE, root mean square error; nRMSE, normalized root mean square error.

**Figure S2**. Variable importance derived from plot-scale random forest models for deadwood carbon density (A) and soil carbon density (B) in the Taohe National Nature Reserve. Relative importance values were normalized within each model, with the most important predictor set to 100%. Volume, stand volume proxy; Northness, north–south component of aspect; Height, mean tree height; Density, stand density; MAP, mean annual precipitation; Soil pH, soil pH in the 0–30 cm layer; and NBR, normalized burn ratio.

**Figure S3**. Cross-validated observed versus predicted carbon density from spatial prediction models for biomass carbon (A), soil carbon (B), litter carbon (C), and ecosystem carbon (D) in the Taohe National Nature Reserve. R^2^, coefficient of determination; RMSE, root mean square error; nRMSE, normalized root mean square error.

**Figure S4**. Variable importance derived from spatial prediction models for biomass carbon density (A), soil carbon density (B), litter carbon density (C), and ecosystem carbon density (D) in the Taohe National Nature Reserve. Relative importance values were normalized within each model, with the most important predictor set to 100%. Soil pH, soil pH in the 0–30 cm layer; Northness, the north–south component of aspect; LAI, leaf area index; MAP, mean annual precipitation; and NBR, normalized burn ratio; NDVI67, the Sentinel-2 NDVI67 median.

**Figure S5**. Pearson correlation matrix among predictor variables included in the plot-scale random forest models. The lower triangle shows pairwise Pearson correlation coefficients, with color intensity indicating the strength and direction of correlations. Asterisks indicate significant correlations (*, *P* < 0.05; **, *P* < 0.01; ***, *P* < 0.001). Volume, stand volume proxy; Height, mean tree height; Basal area, stand basal area proxy; Density, stand density; AI, aridity index; LAI, leaf area index; Soil pH, soil pH in the 0–30 cm layer; Northness, the north–south component of aspect; MAP, mean annual precipitation; NBR, normalized burn ratio; NDVI67, the Sentinel-2 NDVI67 median.

**Table S1.** Sensitivity analysis of biomass carbon and ecosystem carbon prediction excluding stand structural variables.

| Carbon pool | Model | R^2^ | RMSE | MAE | nRMSE % | Predictors |
| --- | --- | --- | --- | --- | --- | --- |
| Biomass | Full model | 0.766 | 16.696 | 13.499 | 19.254 | Elevation; AI; Canopy cover; Age; Density; Height; Basal area; Volume |
|  | Reduced model | 0.353 | 27.226 | 20.999 | 31.398 | Elevation; AI; Canopy cover; Age; Density |
| Ecosystem | Full model | 0.659 | 26.153 | 21.056 | 8.147 | Elevation; AI; Canopy cover; Age; Height; Density; Basal area; Volume |
|  | Reduced model | 0.255 | 38.051 | 28.978 | 11.853 | Elevation; AI; Canopy cover; Age; Density |

Note: R^2^, coefficient of determination; RMSE, root mean square error; MAE, mean absolute error; Bias, mean prediction bias; nRMSE, normalized root mean square error. RMSE, MAE, and Bias are expressed in Mg C ha^-1^. AI, aridity index, calculated as the ratio of mean annual precipitation to potential evapotranspiration; Age, stand age; Height, mean tree height; Density, stand density; Basal area, stand basal area proxy; Volume, stand volume proxy.

**Table S2**. Cross-validated performance of plot-scale multiple linear regression (MLR) models for carbon densities of the forest ecosystem and its component carbon pools in the Taohe National Nature Reserve.

| Carbon pool | R^2^ | RMSE | MAE | Bias | nRMSE % | Predictors |
| --- | --- | --- | --- | --- | --- | --- |
| Biomass | 0.713 | 18.044 | 14.385 | 0.199 | 20.852 | Elevation; AI; Canopy cover; Age; Height; Density; Basal area; Volume |
| Soil | 0.081 | 24.721 | 17.861 | -0.005 | 11.214 | Age; Soil pH; Canopy cover; NBR; MAP |
| Litter | 0.249 | 2.349 | 1.872 | 0.035 | 19.607 | Height; Basal area; Age; LAI; Volume; Density |
| Deadwood | -0.019 | 1.427 | 0.977 | 0.016 | 184.138 | Northness; NDVI67; Age; Density; Height; Volume; Canopy cover |
| Ecosystem | 0.423 | 34.291 | 24.144 | 1.598 | 10.725 | Elevation; AI; Canopy cover; Age; Height; Density; Basal area; Volume |

Note: R^2^, coefficient of determination; RMSE, root mean square error; MAE, mean absolute error; Bias, mean prediction bias; nRMSE, normalized root mean square error. RMSE, MAE, and Bias are expressed in Mg C ha^-1^. AI, aridity index, calculated as the ratio of mean annual precipitation to potential evapotranspiration; Age, stand age; Height, mean tree height; Density, stand density; Basal area, stand basal area proxy; Volume, stand volume proxy; Soil pH, soil pH in the 0–30 cm layer; NBR, normalized burn ratio; MAP, mean annual precipitation; LAI, leaf area index; Northness, the north–south component of aspect; and NDVI67, the Sentinel-2 NDVI67 median.

**Table S3**. Cross-validation performance of spatial prediction models for carbon densities of the forest ecosystem and its component carbon pools in the Taohe National Nature Reserve.

| Carbon pool | mtry | min node size | R^2^ | RMSE | MAE | Bias | nRMSE % | Predictors |
| --- | --- | --- | --- | --- | --- | --- | --- | --- |
| Biomass | 3 | 20 | 0.096 | 38.321 | 30.658 | 0.051 | 50.189 | Soil pH; Northness; LAI |
| Litter | 4 | 20 | 0.042 | 4.500 | 3.388 | -0.081 | 42.921 | Canopy cover; NDVI67; Soil pH; NBR |
| Soil | 1 | 20 | 0.044 | 23.740 | 17.381 | 0.319 | 10.780 | Soil pH; Canopy cover; LAI; MAP |
| Ecosystem | 3 | 20 | 0.144 | 48.869 | 39.359 | -0.137 | 15.880 | Soil pH; Northness; LAI |

Note: Model performance was evaluated using repeated 10-fold cross-validation with 10 repeats. Mtry indicates the number of candidate predictors randomly selected at each split in the random forest model; min node size indicates the minimum number of observations in terminal nodes. R^2^, coefficient of determination; RMSE, root mean square error; MAE, mean absolute error; Bias, mean prediction bias; nRMSE, normalized root mean square error. RMSE, MAE, and Bias are expressed in Mg C ha^-1^. Soil pH, soil pH in the 0–30 cm layer; Northness, the north–south component of aspect; LAI, leaf area index; NDVI67, the Sentinel-2 NDVI67 median variable; NBR, normalized burn ratio; and MAP, mean annual precipitation.

**Table S4**. Species-specific biomass equations used to estimate tree biomass in the Taohe National Nature Reserve.

| Species | Component | a | b | Equation |
| --- | --- | --- | --- | --- |
| *Picea* spp. | Leaf | 0.0717 | 0.6592 | 0.0717 × (DBH² × Height)^0.6592 |
|  | Branch | 0.0132 | 0.8695 | 0.0132 × (DBH² × Height)^0.8695 |
|  | Stem | 0.0518 | 0.8402 | 0.0518 × (DBH² × Height)^0.8402 |
|  | Root | 0.0279 | 0.7974 | 0.0279 × (DBH² × Height)^0.7974 |
| *Abies* spp. | Leaf | 0.005 | 0.9085 | 0.005 × (DBH² × Height)^0.9085 |
|  | Branch | 0.0179 | 0.862 | 0.0179 × (DBH² × Height)^0.862 |
|  | Stem | 0.0342 | 0.8812 | 0.0342 × (DBH² × Height)^0.8812 |
|  | Root | 0.0094 | 0.8824 | 0.0094 × (DBH² × Height)^0.8824 |
| *Cupressus funebris* | Leaf | 0.0126 | 0.8808 | 0.0126 × (DBH² × Height)^0.8808 |
|  | Branch | 0.0089 | 1.0449 | 0.0089 × (DBH² × Height)^1.0449 |
|  | Stem | 0.0239 | 0.9716 | 0.0239 × (DBH² × Height)^0.9716 |
|  | Root | 0.0616 | 0.8471 | 0.0616 × (DBH² × Height)^0.8471 |
| *Populus davidiana* | Leaf | 0.0291 | 0.6136 | 0.0291 × (DBH² × Height)^0.6136 |
|  | Branch | 0.0036 | 0.8437 | 0.0036 × (DBH² × Height)^0.8437 |
|  | Stem | 0.0859 | 0.8001 | 0.0859 × (DBH² × Height)^0.8001 |
|  | Root | 0.0143 | 0.8772 | 0.0143 × (DBH² × Height)^0.8772 |
| *Betula platyphylla* | Leaf | 0.0054 | 0.7486 | 0.0054 × (DBH² × Height)^0.7486 |
|  | Branch | 0.0165 | 0.7471 | 0.0165 × (DBH² × Height)^0.7471 |
|  | Stem | 0.0642 | 0.8734 | 0.0642 × (DBH² × Height)^0.8734 |
|  | Root | 0.0042 | 1.0629 | 0.0042 × (DBH² × Height)^1.0629 |
| *Larix* spp. | Leaf | 0.005 | 0.7309 | 0.005 × (DBH² × Height)^0.7309 |
|  | Branch | 0.0028 | 0.9417 | 0.0028 × (DBH² × Height)^0.9417 |
|  | Stem | 0.0334 | 0.9171 | 0.0334 × (DBH² × Height)^0.9171 |
|  | Root | 0.0226 | 0.852 | 0.0226 × (DBH² × Height)^0.852 |
| *Quercus* spp. | Leaf | 0.0346 | 0.6056 | 0.0346 × (DBH² × Height)^0.6056 |
|  | Branch | 0.0079 | 1.001 | 0.0079 × (DBH² × Height)^1.001 |
|  | Stem | 0.0236 | 0.9681 | 0.0236 × (DBH² × Height)^0.9681 |
|  | Root | 0.0549 | 0.8138 | 0.0549 × (DBH² × Height)^0.8138 |
| Broad-leaved trees | Leaf | 0.0022 | 0.8595 | 0.0022 × (DBH² × Height)^0.8595 |
|  | Branch | 0.0011 | 1.1909 | 0.0011 × (DBH² × Height)^1.1909 |
|  | Stem | 0.021 | 0.9642 | 0.021 × (DBH² × Height)^0.9642 |
|  | Root | 0.053 | 0.7452 | 0.053 × (DBH² × Height)^0.7452 |
| Coniferous trees | Leaf | 0.0258 | 0.7037 | 0.0258 × (DBH² × Height)^0.7037 |
|  | Branch | 0.0128 | 0.8916 | 0.0128 × (DBH² × Height)^0.8916 |
|  | Stem | 0.0427 | 0.8545 | 0.0427 × (DBH² × Height)^0.8545 |
|  | Root | 0.0079 | 0.9385 | 0.0079 × (DBH² × Height)^0.9385 |

Note: Biomass equations were obtained from Zhou et al. (2018).
